# Longitudinal Network Structure and Changes of Clinical Risk and Protective Factors in a Nationwide Sample of Forensic Psychiatric Patients

**DOI:** 10.1177/0306624X20923256

**Published:** 2020-05-29

**Authors:** Stefan Bogaerts, Marinus Spreen, Erik Masthoff, Marija Jankovic

**Affiliations:** 1 Tilburg University, the Netherlands; 2 Fivoor Science and Treatment Innovation (FARID), Rotterdam, the Netherlands; 3 NHL Stenden University of Applied Sciences, Leeuwarden, the Netherlands

**Keywords:** network analysis, clinical risk factors, clinical protective factors, the HKT-R, the risk–need–responsivity model, the good lives model

## Abstract

In this study, we investigated network configurations of 14 Clinical risk and protective factors in a sample of 317 male forensic psychiatric patients across two time points: at the time of admission to the forensic psychiatric centers (T1) and at the time of unconditional release (T2). In terms of network structure, the strongest risk edge was between “hostility–violation of terms” at T1, and between “hostility–impulsivity” at T2. “Problem insight–crime responsibility” was the strongest protective edge, and “impulsivity–coping skills” was the strongest between-cluster edge, at both time points, respectively. In terms of strength centrality, “cooperation with treatment” had the highest strength centrality at both measurement occasions. This study expands the risk assessment field toward a better understanding of dynamic relationships between individual clinical risk and protective factors and points to the highly central risk and protective factors, which would be the best for future treatment targets.

In the past decades, a large amount of research has been done on dynamic risk factors (DRFs) that are related to criminal behavior and reoffending. Originally, supported by general personality models, cognitive social learning perspectives, and the risk–need–responsivity model (RNR: Andrews & Bonta, 2006; Andrews et al., 2012), eight risk factors (Central Eight) were identified, which are prominent in the explanation and prediction of criminal behavior, treatment outcome, and reoffending. These factors were the Big Four risk factors (history of antisocial behavior, antisocial personality pattern, antisocial cognition, and antisocial peers) and the Moderate Four risk factors (family/marital condition, school/work, leisure/recreation, and substance abuse). The Central Eight risk factors have played subsequently a crucial role in the development of various risk assessment tools and are aimed to predict the likelihood that a prisoner or forensic psychiatric patient will reoffend in the same offense or another one after release. Furthermore, these tools are also used to investigate the treatment progress of forensic patients during their stay in the institution and to estimate the likelihood of future inpatient violence (Jeandarme et al., 2019). These predictions are based on historical, clinical, and future factors, and predictions can be made at scale level and/or at factor level.

Although the RNR model is currently a dominant treatment approach in the forensic field, it has been recently criticized for overemphasizing offender risk factors and not paying enough attention to protective factors of offenders (Ward et al., 2007). The good lives model (GLM) has been therefore proposed as an alternative or addition to the RNR approach. The GLM is a strength-based approach to the rehabilitation of offenders that focuses primarily on increasing competencies and skills for offenders to, indirectly, reduce the risk of reoffending (Höing et al., 2013). According to this model, motivating offenders and creating a sound therapeutic alliance are key components of effective treatment (Ward & Brown, 2004). The purpose of this study was to get more insight into which factors, that is, risk, protective, or both, should be the pivotal targets of treatment options in forensic psychiatric patients measured at the moment of admission and unconditional release.

Furthermore, a major limitation of how risk and protective factors are used today in research and clinical work is that only information is available about the linear independent contribution of individual risk/protective factors or (sub)scales in the prediction of the risk of recidivism (Fazel et al., 2012). Information about unidirectional or bidirectional associations between single risk and protective factors, and whether associations between factors can change over time, is currently hardly applied in forensic psychiatry. Because high rates of comorbidities have been reported in forensic psychiatric patients and are thought to contribute to reoffending (Black et al., 2010), it is very important to gain insight into the interaction of risk and protective factors, and changes in these interactions during inpatient forensic treatment.

Relying only on linear and independent associations of risk and protective factors, and not considering reciprocal associations between risk and protective factors may attenuate or mask significant information, which cannot be taken into account in the treatment process (Beggs, 2010). Hence, it is important to study associations at the item level and to explore how DRFs and protective factors are reciprocally associated (Gabrielle et al., 2016). Therefore, this study aimed to investigate changes in the seriousness of the risk and protective factors at the time of admission compared with the moment of unconditional release. To address associations between factors, network analysis may help researchers and clinicians to better comprehend these complex dynamic associations and help clinicians to decide, which factors are most important to focus on, in a broader set of risk and protective factors. To our knowledge, no studies on network associations and configuration of risk and protective factors in forensic psychiatric patients have been published so far. Some recent forensic studies have conducted research on network configurations in psychopathy, investigating the structure of core symptoms of psychopathic personality disturbance (McCuish et al., 2019; Preszler et al., 2018; Verschuere et al., 2018).

## A Network Approach to Dynamic Risk and Protective Factors

Even though latent variable models are widely used in psychological research, the validity of these models has been recently challenged in the literature. For example, Markus and Borsboom (2013) suggested that network analysis may be more efficient for the study of psychological attributes, as it is closer to reality than traditional models entailing latent variables (Borsboom & Cramer, 2013). Hence, Borsboom and Cramer (2013) developed a network approach to analyze causal associations (strength) among symptoms in which manifest measured symptoms or risk factors are the basis of their approach. A causal system of dynamically interacting risk and protective factors allows researchers to study significant associations between a set of individual factors and demonstrates which factors have the greatest impact and influence on other factors. A variable with the highest influence is called the most central. That is, if a symptom (or risk/protective factor) has many connections to other symptoms in a network, it is likely that this symptom will also affect the evolution of other symptoms. Longitudinally, it can be studied in what way associations between a set of risk and protective factors evolve over time and whether the same factors remain central. Also, the proposed network approach may provide insight into the overall connectedness of risk and protective factors. Networks with more strongly connected risk factors are more prone to future inpatient violence and/or reoffending (Fried et al., 2017).

## The Present Study

In this study, a set of individual risk and protective factors is regarded to be part of the same network in which its positions in the structure are reinforced or weakened by each other through positive or negative feedback loops that are not exchangeable (central vs. peripheral). To illustrate, consider a forensic male patient who has been in a highly secured psychiatric institution for 3 years. He was told that an external leave assessment advisory body rejected his request for unguided leave. This was very disappointing for him and caused a lot of frustration and anger. As a reaction, he held the staff responsible for the rejection and projected feelings of hostility toward the staff. In this period, he also temporarily stopped cognitive behavioral treatment and violated some rules by displaying verbally aggressive behavior against fellow patients and staff, and refusing to perform daily tasks, such as cleaning the kitchen. In this fictional case, some risk factors can be viewed as interconnected and may reinforce each other negatively. The rejection apparently negatively influences his feelings (hostility) and his treatment progress (noncompliance), which makes him rebellious (violation of rules). Various studies have shown that these risk factors may increase the risk of violent aggression and/or reoffending, and protective factors can buffer or reduce the likelihood of aggression and also can have an effect on risk factors (Schuringa et al., 2019). By analyzing the network of interconnections at the time of admission and unconditional release, it can be made transparent how these factors influence each other and which of these activated factors are the most central and influenceable. In network analysis, risk and protective factors are represented as nodes and the associations between pairs of risk and protective factors are depicted as edges (Borsboom & Cramer, 2013; Preszler et al., 2018).

To summarize, this longitudinal study aimed to investigate the network configuration and centrality indices of risk and protective factors based on the 14 Clinical factors of the Historical Clinical Future-Revised (HKT-R) in a sample of forensic psychiatric patients. We analyzed the network configuration and centrality indices at the time of admission and whether any changes occurred within associations and centrality indices at the time of unconditional release. Based on the RNR and GLM models (Andrews & Bonta, 2006; Ward et al., 2007), we hypothesized that risk factors would have a more central position at T1 compared with T2, whereas protective factors would have a more central position at T2 compared with T1.

## Method

### Participants and Institutions

The initial study sample consisted of 347 patients with TBS orders who were released unconditionally between 2004 and 2008 from any of the 12 Dutch forensic psychiatric centers. Thirty female offenders were excluded as the HKT-R was only validated in a male TBS (Ter Beschikking Stelling) population. The final sample consisted of 317 male patients. The Dutch inpatient forensic psychiatry has three security levels (from highest to least secured: forensic psychiatric centers (FPCs), forensic psychiatric clinics (FPKs), and forensic psychiatric units (FPAs)). The forensic facility to which an offender is referred depends on the presence and severity of clinical DRFs, the intensity of the required mandatory treatment, and the risk of reoffending (high, moderate, or low). Offenders, who are assigned to a FPC, have been sentenced to a TBS measure. A TBS order can be imposed for forensic patients who have committed a serious crime caused by a severe mental illness during or a severe personality disorder (Van Marle, 2002).

### Procedure

The demographic, clinical, and criminal data were retrospectively derived from the electronic criminal and patients’ files. These files consisted of information on the patients’ background and criminal history, psychiatric evaluation reports, treatment plans, leave requests, and prolongation advice. Twenty intensively trained research assistants coded the HKT-R for five time points (juridical psychiatric observation, admission in the FPC, approval of unguided leave, conditional release, and unconditional release). For information about predictive validity of the HKT-R, we refer to previous research (Bogaerts et al., 2018; Spreen et al., 2014). In this study, the same dataset was used and two time points were studied: admission to the forensic institution and unconditional release from the institution. Ethical permission was given by the Scientific Research Committee of FPC Kijvelanden. Also, permission for this study was given by the Dutch Ministry of Security and Justice and by the directors of the 12 FPCs involved in the study (Bogaerts et al., 2018; Spreen et al., 2014). The dataset was made available to the researchers anonymously, and the data could not be traced to individual patients.

### Instrument

The HKT-R (Spreen et al., 2014) is a structured professional tool for assessing the risk of violent recidivism in forensic psychiatric patients. The HKT-R consists of 33 factors spread over three domains: 12 Historical, 14 Clinical, and seven Future factors. All factors are rated on a 5-point scale, ranging from 0 to 4, in which “0” represents *no risk* and “4” represents a *high level of risk*. The Historical domain relates to the offender’s personal history up to the moment of the arrest for the current index-offense (e.g., judicial history, employment history, and victim type). The Clinical domain contains 14 factors that are divided into seven risk (e.g., impulsivity and hostility) and seven protective factors (e.g., coping skills and cooperation with treatment). The Clinical domain refers to the offender’s behavior in the last 12 months (e.g., problem insight, psychotic symptoms, and antisocial behavior). In our study, all protective factors were recoded so that higher scores indicated higher protection against reoffending (“0” represents *no protection* and “4” represents *high protection*). The Future domain is related to the assessment of potential risks, which could emerge after discharge from the FPC (e.g., stressful circumstances, living arrangements, and work situation).

For patients with a TBS order, a risk assessment of the Clinical items must be performed at least once a year. The annual scores on the 14 Clinical factors indicate whether a reduction in risk factors and/or an improvement in protective factors has occurred, compared with the previous 12 months of stay in the institution. Hence, if changes occur, that could assumingly be ascribed to the given treatment. In this study, only the Clinical dynamic factors were included as Historical factors are static and irreversible, and Future factors are exclusively related to the situation after release. Internal consistency for the Clinical domain was good at both measurement points, with Cronbach’s α being α_T1_ = .80 and α_T2_ = .83, respectively. Descriptive statistics of the clinical risk and protective factors are presented in Table 1.

**Table 1.** Means and Standard Deviations of the Clinical HKT-R Items at Both Measurement Occasions.

Clinical HKT-R factors (range 0–4)	M (*SD*) at each time point		Test statistics	*p*
	T1	T2		
1. Psychotic symptoms	0.33 (0.7)	0.13 (0.4)	*t*(272) = −5.530	<.001
2. Addiction	0.55 (1.1)	0.42 (1.0)	*t*(273) = −1.469	.143
3. Impulsivity	2.05 (1.3)	0.95 (1.1)	*t*(221) = −12.949	<.001
4. Antisocial behavior	1.39 (1.4)	0.51 (1.0)	*t*(228) = −10.030	<.001
5. Hostility	1.39 (1.1)	0.49 (0.8)	*t*(208) = −11.875	<.001
6. Violation of terms and agreements	1.03 (1.4)	0.38 (0.9)	*t*(276) = −6.853	<.001
7. Influence of risky network-members	0.16 (0.6)	0.07 (0.3)	*t*(248) = −3.505	.003
8. Problem insight	1.49 (1.0)	2.85 (1.0)	*t*(222) = 17.248	<.001
9. Social skills	1.98 (0.9)	3.12 (1.0)	*t*(236) = 18.438	<.001
10. Self-reliance	3.53 (0.9)	3.69 (0.7)	*t*(241) = 3.572	<.001
11. Cooperation with treatment	2.54 (1.2)	3.37 (1.0)	*t*(272) = 10.608	<.001
12. Responsibility for the offense	1.77 (1.1)	2.60 (1.3)	*t*(133) = 7.926	<.001
13. Coping skills	1.31 (0.9)	2.73 (1.0)	*t*(226) = 18.798	<.001
14. Labor skills	2.88 (1.3)	3.44 (0.9)	*t*(206) = 5.933	<.001

Note. HKT-R = Historical Clinical Future-Revised.

### Statistical Analyses

The data were analyzed with SPSS v.24.0 (IBM Corp., Armonk, NY, USA) and the free software environment R (R Core Team, 2016). First, we performed missing data imputation, and second, we performed network estimation, network inference, and network parameter accuracy.

#### Missing data imputation

To preserve power, missing values on Clinical HKT-R factors were imputed with the Multivariate Imputation by Chained Equations (MICE) R-package (van Buuren et al., 2015). The MICE algorithm uses information from other variables in a dataset to predict and impute the missing data (see Buuren & Groothuis-Oudshoorn, 2010, for more detail). In this study, missing values (28.6%) were predicted by using into account all clinical HKT-R variables.

#### Network estimation

For both time points, the network of associations was estimated with the Gaussian graphical model (GGM; Costantini et al., 2015) using the R-package qgraph (Epskamp et al., 2012). The GGM is a network analysis technique for continuous or ordinal normally distributed data in which nodes represent variables and the edges partial correlations between the variables (Costantini et al., 2015). Every edge represents a connection between two variables after conditioning on all other variables in the network. As the GGM assumes that data are normally distributed, non-normally distributed Clinical HKT-R items were transformed utilizing the Henze–Zirkler’s test (Henze & Zirkler, 1990) with the Multivariate Normality Test (MVN) R-package (Korkmaz et al., 2014). The Henze–Zirkler’s test is based on a nonnegative functional distance that measures the distance between two distribution functions (Korkmaz et al., 2014). In the GGM, a large number of parameters needs to be estimated (e.g., this network of 14 nodes requires the estimation of 105 parameters), which may result in some false positive edges. We employed the least absolute shrinkage and a selection operator (LASSO; Tibshirani, 1996) in combination with the extended Bayesian information criterion (EBIC) model selection (Chen & Chen, 2008) to control for false positive edges that may arise due to multiple testing. This procedure is based on a regularizing penalty, which limits the total number of edges in the network by shrinking small edge values to exactly zero. The result is a sparse network with as few edges as possible (i.e., parsimonious). The graph was plotted by a “spring” layout that posits more strongly connected nodes closer to each other (Fruchterman & Reingold, 1991). Green edges indicate positive associations, and red edges indicate negative associations. The thicker the edge, the stronger the association between two nodes is.

#### Network inference

To gain more insight into which nodes are the most important in the network, centrality analysis was performed with the bootnet R-package (Epskamp & Fried, 2017). Centrality estimates the importance of a node or edge for the connectivity of the network. We calculated *strength* for each node, which represents a sum of all associations a given node has with all other nodes (Costantini et al., 2015). The *strength* has always been the most accurately estimated centrality measure, while other centrality indices, such as *betweenness* and *closeness* only reach the threshold for reliable estimation in large or very large samples (Epskamp & Fried, 2018). Node *strength* was presented as standardized *z* scores. The greater the *z* score, the more central the node is.

The network parameter accuracy was estimated with the R-package bootnet (Epskamp & Fried, 2017). The stability of edge was calculated with *bootstrapping* (Epskamp et al., 2018). The procedure relies on random sampling with replacement of the original data, followed by estimating the edge weights based on the samples. This results in a bootstrapped 95% confidence interval (CI) with bounds defined by Epskamp et al. (2018) as follows: taking the interval between quantiles 1/2α and 1 − 1/2α of the bootstrapped values. That is, in 95% of the cases, the bootstrapped CIs will contain the true value of the parameter (Epskamp et al., 2018).

The stability of centrality measure was based on *subsetting* (Epskamp et al., 2018). This procedure determines how many cases can be dropped from the original data before the results become unstable. Furthermore, to quantify the stability of centrality measure, we also computed the correlation stability coefficient (CS-coefficient). The CS-coefficient represents the maximum number of cases that can be removed from the data to retain, with 95% probability, a correlation of .70 or higher between original centrality measure and the centrality of networks computed with fewer cases (Epskamp et al., 2018). A CS-coefficient larger than .25 is considered “somewhat” stable and above .50 “stable.” Finally, we estimated whether nonzero edge weights or centrality indices differ significantly from one another with the bootstrapped difference test (Epskamp et al., 2018), using 1,000 bootstrap samples and α = .05. It takes the difference of bootstrap values between all pairs of edges or centrality indices and reveals a bootstrapped CIs around the estimated difference scores. The inspection of zero being in the bootstrapped CIs is a valid null-hypothesis test (Epskamp et al., 2018). Only the edge-weights/centrality measures that were significantly higher from most other edges/centrality measures in the network were interpreted.

## Results

### Sample Characteristics

The sample in this study consisted of male forensic psychiatric patients (*n* = 317). Of this sample, 95.3% (*n* = 302) had the Dutch nationality, 2.7% (*n* = 9) had a different nationality, and 2% (*n* = 6) had another nationality in addition to the Dutch. The majority of patients received both a TBS order and prison sentence (*80*%, *n* = 254). The mean age at admission to the FPCs was 31.6 years (*SD* = 8.14, range = 16.32–64.81). The index offenses included manslaughter (*n* = 120, 37.9%), murder (*n* = 52, 16.4%), sexual violence against adults (*n* = 57, 18.0%), sexual violence against minors (*n* = 23, 7.3%), arson (*n* = 45, 14.2%), robbery (*n* = 125, 39.4%), moderate violence (*n* = 193, 60.9%), and severe violence (*n* = 86, 27.1%). Patients could be convicted of multiple index offenses at the same time. At the beginning of treatment, the most frequent *Diagnostic and Statistical Manual of Mental Disorders* (4th ed.; *DSM-IV*; American Psychiatric Association, 1994) diagnoses were personality disorder not otherwise specified (PDNOS; *n* = 166, 52.4%), Cluster B personality disorders (*n* = 84, 26.5%), substance-related disorders (*n* = 133, 35.5%), and schizophrenia and other psychotic disorders (*n* = 70, 22.1%). These percentages do not count to 100% because in most patients, comorbid disorders were present. The mean total IQ in the study sample was 98.20 (*SD* = 15.62, range 52–139). After receiving treatment for a mean period of 8.02 years (*SD* = 3.34, range 2.63–27.68), the patients were unconditionally discharged from the forensic psychiatric hospital at a mean age of 40.14 years (*SD* = 3.18, range 2.63–27.68). The violent recidivism rate in this sample of forensic patients was 20.7% (*n* = 36) within 5 years following release (Bogaerts et al., 2018).

### Description of the Clinical Factors on T1 and T2

As can be seen from Table 1, risk factors with the highest mean at both time points were impulsivity, antisocial behavior, and hostility. Protective factors with the highest mean at both time points were self-reliance, labor skills, and cooperation with treatment. A *t* test for dependent samples revealed that all protective factors significantly increased from T1 to T2, whereas all risk factors significantly decreased from T1 to T2, except addiction, which also decreased but not significantly (see Table 1). The risk factors which decreased the most from T1 to T2 were impulsivity, hostility, and antisocial behavior, whereas the protective factors which increased the most from T1 to T2 were coping skills, social skills, and problem insight. Overall, we can notice less problematic and more protective behavior at T2 than at T1.

### Network Structure

**Figure 1.**
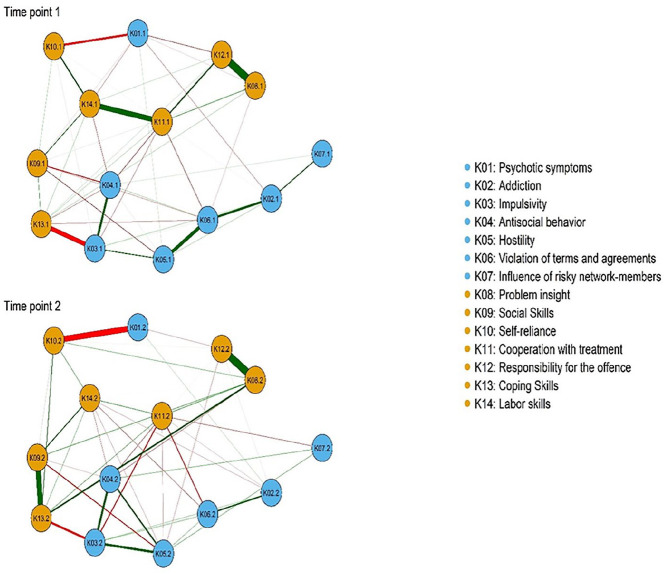
Network structure of clinical risk and protective factors at admission (T1) and discharge (T2). *Note.* Orange circles refer to protective factors and blue circles refer to risk factors. Green lines indicate positive associations and red lines indicate negative associations. The thicker the line, the stronger the association between two nodes is.

The estimated networks of associations of the 14 risk and protective factors at admission (T1) and discharge (T2) are shown in Figure 1. Approximately, 48% of all edges in the network were set to zero at T1, and 49% at T2, respectively. Overall, associations within clusters were positive, while between clusters were negative.

At T1, within protective factors, the strongest edges were between problem insight and crime responsibility (K08–K12) and between cooperation with treatment and labor skills (K11–K14). Thus, cooperation with treatment was moderately associated with crime responsibility (K11–K12), whereas labor skills was moderately associated with self-reliance (K10–K14). Within risk factors, the strongest edge was between hostility and violation of terms (K05–K06). Violation of terms was in turn moderately associated with addiction (K06–K02). There was also a strong edge between impulsivity and antisocial behavior (K03–K04). Considering the associations between clusters, the strongest negative edge was between impulsivity and coping skills (K03–K13). There was also a moderate negative edge between psychotic symptoms and self-reliance (K01–K10), and a moderate to a weak edge between antisocial behavior and social skills (K04–K09). These results were supported by our network parameter accuracy analysis (i.e., the bootstrapped difference test; see Figure A1 in the Supplemental Appendices).

Based on the bootstrapped difference test (see Figure A2 in the Supplemental Appendices), at T2, only six edges were significantly larger than all other edges in the network. Within protective factors, the edge between problem insight and crime responsibility (K08–K12) remained the strongest edge in the network. There was also a strong edge between social skills and coping skills (K09–K13). Within risk factors, hostility was moderately associated with impulsivity (K05–K03) and somewhat less strongly associated with antisocial behavior (K05–K04). When it comes to the associations between clusters, the edges between impulsivity and coping skills (K03–K13) and between psychotic symptoms and self-reliance (K01–K10) continued to be the strongest edges at T2, as well.

### Centrality Indices

**Figure 2.**
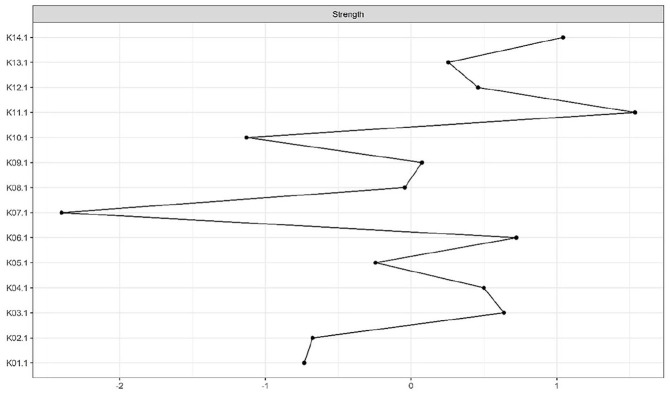
Strength centrality index at T1. *Note.* Strength centrality index is presented as standardized *z* score. The greater the *z* score, the more central the risk factor is.

**Figure 3.**
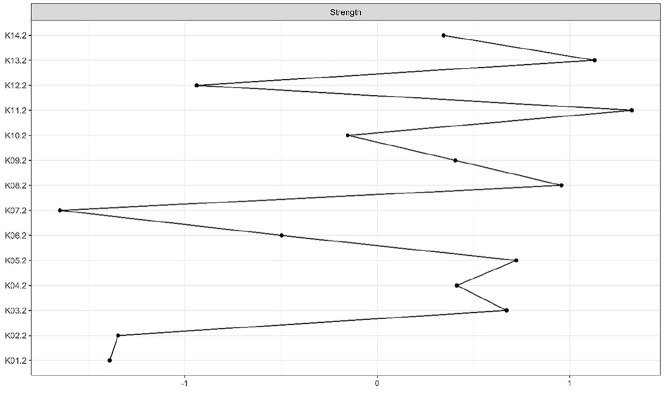
Strength centrality index at T2. *Note.* Strength centrality index is presented as standardized *z* score. The greater the *z* score, the more central the risk factor is.

According to the CS-coefficient, the strength centrality index was considered stable at both measurement occasions. The node with the highest standardized strength centrality was cooperation with treatment (K11) at both time points (see Figures 2 and 3). The bootstrapped difference test for centrality indices indicated that cooperation with treatment (K11) had significantly higher strength than most other nodes at T1 and T2 (see Figures A7 and A8 in the Supplemental Appendices).

## Discussion

This study is the first to investigate network configuration of risk and protective factors based on the 14 Clinical factors of the HKT-R, in a sample of forensic psychiatric inpatients, over two time points. Also, highly central risk and protective factors were investigated at the time of admission to the FPCs and whether any changes occurred with regard to centrality at the time of unconditional release from the FPCs. We hypothesized that crime-related risk factors would have a more central position at T1, while protective factors would be more central at T2. Overall, the results suggested that not all clinical factors were equally pronounced at both time points, supporting the previous notion that treating risk and protective factors as a homogeneous group might be naive (Gabrielle et al., 2016). In terms of network structure, we found that all associations within clusters were positive, and between clusters were negative. In terms of centrality, the protective factor cooperation with treatment had the highest standardized strength centrality in the network at both time points.

### Network Configuration of Risk and Protective Factors

Although our network model does not allow inferences regarding the (causal) directionality of pathways between nodes, our findings fit well into a growing number of publications, and therefore we interpreted significant edges in line with previous research (Verschuere et al., 2018).

At the time of admission to the FPCs (T1), we found that 10 edges were significantly stronger than most other edges in the network. Of these, the strongest protective edges were “problem insight–crime responsibility” and “cooperation with treatment–labor skills.” The strongest risk edges were “hostility–violation of terms” and “impulsivity–antisocial behavior.” There were also moderate edges between “cooperation with treatment–crime responsibility,” and between “labor skills–self-reliance” within protective factors, and between “addiction–violation of terms” within risk factors. All the associations within the clusters were positive, which implies from a network perspective that they are thought to reinforce each other through positive feedback loops (Borsboom & Cramer, 2013). As expected, there were also negative associations between protective and risk factors. Specifically, we found negative moderate edges between “antisocial behavior–social skills,” “psychotic symptoms–self-reliance,” and “impulsivity–coping skills.”

A possible explanation for our findings could be that patients who lack problem insight or the awareness of individual pitfalls are more likely to have the impaired capacity for taking crime responsibility, which could lead to poorer treatment adherence. In addition, less involvement in the treatment progression along with deficiencies in the realm of self-reliance could further lessen a patient’s ability to adequately perform work or labor activities. Having (a) psychotic episode(s) could disable a patient’s ability to complete essential daily tasks independently. The results also suggested that patients who were more impulsive (i.e., who behave in an unpredictable and/or thoughtless manner) were also more prone to use maladaptive coping strategies and develop antisocial behavior, which could also arise due to lack of social skills. Similarly, addiction problems, as well as hostility, could trigger a commission of more severe breaches in the secured forensic settings (e.g., severe verbal and/or physical aggression, drug dealing, withdrawal from supervision).

Interestingly, at the time of unconditional release (T2), there was a substantial decrease in the number of significant edges. Specifically, only six edge-weights were significantly higher than most other edges in the network. Within protective factors, the edge between “problem insight–crime responsibility” was also the strongest edge in this network, replicating our finding from T1. Interestingly, at T2, a strong protective edge emerged between “social skills–coping skills.” Within risk factors, at T2, impulsivity was moderately associated with hostility and antisocial behavior, respectively. Furthermore, the edges between “impulsivity–coping skills” and between “psychotic symptoms–self-reliance” were replicated at T2, as well. The novel findings suggested that frequent and/or severe impulsive behavior could lead to more signs of hostility and antisocial behavior, whereas encountering problems due to insufficient social skills could trigger the use of maladaptive coping strategies.

Our findings are in line with previous studies showing that impulsivity leads to use of maladaptive coping strategies (Bornovalova et al., 2005; Kunst et al., 2010, 2011; Vollrath & Torgersen, 2000), development of antisocial behavior (Fornells et al., 2002; Maneiro et al., 2017; Moeller et al., 2001), and hostility and aggression (Bresin, 2019; Scarpa, & Raine, 2000). In addition, our findings also found support in previous studies showing that psychotic patients were characterized with impaired everyday functioning (Klapow et al., 1997; Viertiö et al., 2012) and that patients who were not able to satisfactorily maintain social contacts with his life- and work-environment were more prone to use maladaptive coping strategies (Brunt & Hansson, 2002; Van Der Horst et al., 2010) and to develop antisocial behavior (Coie & Dodge, 1998; Farrington & Loeber, 2001; Lösel & Beelmann, 2003). To support our novel findings, further investigation and replications in larger samples are needed.

Overall, results showed that there were far more significant edge-weights at T1, especially among crime-related risk factors, than at T2. This is in line with the RNR model (Andrews & Bonta, 2006). Indeed, crime-related risk factors are more directly related to offending and are therefore expected to be more pronounced at the time of admission to the FPCs than at the time of unconditional release. This is further supported by the results of the t test for dependent samples, which showed that all clinical risk factors significantly decreased on average between T1 and T2, except addiction, which also decreased but not significantly, making a level of risk factors at the second measurement occasion less problematic. A linear decrease in DRFs over time was demonstrated in previous research, as well (Douglas et al., 2011).

### Centrality

The protective factor cooperation with treatment was the most central factor among all clinical factors at both time points. To the best of our knowledge, from a network perspective, therapeutic interventions would be more effective if they target more central nodes. Therefore, improving treatment compliance and treatment readiness would probably lead to an overall reduction in the likelihood of reoffending. This finding is in line with previous studies, conducted in a sample of delinquent adolescents and adults, which showed that compliance with treatment and treatment quality can reduce the risk, whereas noncompliance with treatment can increase the risk of future recidivism (Sturgess et al., 2016).

Consistent with the RNR and GLM model, we found that protective factors (i.e., cooperation with treatment) were more influential than crime-related risk factors at T2. However, contrary to our expectations, we did not find that crime-related risk factors would be more influential than protective factors in the network at T1. Other forensic psychiatric network studies, though in the field of psychopathy (McCuish et al., 2019; Preszler et al., 2018; Verschuere et al., 2018), also showed that antisocial facets (i.e., crime-related risk factors in our study) have low centrality. Furthermore, even though the RNR model does not diminish the importance of targeting (non-)criminogenic needs (e.g., protective factors), it however focuses primarily on the detection and modification of criminogenic needs (i.e., DRFs). In contrast, the GLM (Ward et al., 2007) concentrates more on increasing competencies and skills for offenders to indirectly reduce the risk of reoffending. According to this model, a treatment readiness of offenders is a function of both internal (person) and external or contextual factors (Ward & Brown, 2004), and addressing (non-)criminogenic needs has an important role in enhancing offender’s motivation and creating a more effective therapeutic environment for the offender. Therefore, our results fit better in this strengths-based model and give support to the GLM, although partly support the RNR model, as well (Ward et al., 2007).

### Limitations and Future Directions

Some limitations should be considered while interpreting the results of this study. First, the estimation of psychological networks requires very large sample sizes, as many parameters are to be estimated, and therefore our results based on a sample size of 317 patients should be considered exploratory (more details on sample sizes in network analysis could be found elsewhere; Epskamp & Fried, 2018). Thus, the generalizability of these findings is limited to the population of male forensic inpatients. Future research should attempt to replicate our findings in multiple large datasets of forensic psychiatric inpatients. Next, in this study, we measured how risk and protective factors changed within two time points. Even though this could help us get more insight into the change of factors, that is, from admission to the forensic clinic to unconditional release (i.e., increasing or decreasing), considering multiple time-points in future studies could even further extending our knowledge about different patterns and trajectories of change (Hendry, 2013). This is important for establishing if meaningful progress is being made against set treatment targets. Finally, we calculated the edge-weights precluding the estimations of important network features, such as autoregressive and cross-lagged pathways.

### Clinical Implications

It is important to stress the clinical added value of this study. First, the network approach offers a new way of understanding interconnections between clinical risk and protective factors at different measurements during a stay of forensic patients in FPCs. This method emphasizes functional interconnections (edges) and provides insight into underlying associations and mutual influencing of clinical risk and protective factors (Fried & Nesse, 2015). Second, the added value of using a longitudinal design allows researchers and clinicians to interpret changes in network connections at different time points, both visually and statistically. Through graphic representations, the clinician can easily gain knowledge and insight into which risk and protective factors are related (at the group level). This knowledge about which associations between risk/protective factors are important and how they can change over time could help clinicians modify risk and protective factors and give offenders more protection from the occurrence of reoffending. Third, the network approach may also contribute to differential diagnostics. For example, we can consider a person who committed a crime as a result of an acute psychotic disorder. The traditional approach will immediately recommend antipsychotic medication. However, the network approach will provide additional information of which symptoms, both risk factors and protective factors, are active and how they are related to one other, to obtain a broader diagnostic profile regarding stabilizing or offense-enhancing factors. For example, when this person actively participated in the treatment, it is more likely that a future crime is being buffered.

## Conclusion

This study is the first to report the network of dynamic risk and protective factors in a sample of forensic psychiatric patients between two time points. Our findings give support to both models, the RNR and GLM, signifying that they should be viewed as complementary rather than opposing, and by highlighting the merits of each, offender rehabilitation could be maximized. This work expands the risk assessment field toward a better understanding of the complex dynamic relationships between many individual clinical risk and protective factors and points to the highly central clinical factors, which would be the best candidates for future treatment targets.

## Supplemental Material

Appendices – Supplemental material for Longitudinal Network Structure and Changes of Clinical Risk and Protective Factors in a Nationwide Sample of Forensic Psychiatric Patients Click here for additional data file. Supplemental material, Appendices for Longitudinal Network Structure and Changes of Clinical Risk and Protective Factors in a Nationwide Sample of Forensic Psychiatric Patients by Stefan Bogaerts, Marinus Spreen, Erik Masthoff and Marija Jankovic in International Journal of Offender Therapy and Comparative Criminology
