# Supplementary material for: Longitudinal Network Structure and Changes of Clinical Risk and Protective Factors in a Nationwide Sample of Forensic Psychiatric Patients
Source: Int J Offender Ther Comp Criminol. 2020 May 29;64(15):1533–50. doi: 10.1177/0306624X20923256 (PMC7495686; doi:10.1177/0306624X20923256)
Supplement: Appendices – Supplemental material for Longitudinal Network Structure and Changes of Clinical Risk and Protective Factors in a Nationwide Sample of Forensic Psychiatric Patients [file Appendices.pdf]

Appendices

Table A1

Correlations between T1 and T2

Variables	*r*

1. Psychotic symptoms	.574**
2. Addiction	.207**
3. Impulsivity	.552**
4. Antisocial behavior	.608**
5. Hostility	.433**
6. Violation of terms and agreements	.236**
7. Influence of risky network-members	.360**
8. Problem insight	.420**
9. Social Skills	.501**
10. Self-reliance	.609**
11. Cooperation with treatment	.328**
12. Responsibility for the offence	.580**
13. Coping Skills	.320**
14. Labor skills	.365**

Note. ** Correlation is significant at the 0.01 level (2-tailed).

Table A2

Description of clinical risk and protective factors (Spreen et al, 2014)

Risk factors	Description of clinical HKT-R factors
K01: Psychotic symptoms	• The extent to which the patient showed active psychotic symptoms such as delusions (e.g.,

	hearing voices). Particular attention should be given to symptoms or delusions containing aspects of violence, paranoid content and/or with aspects of executing control over fear.
K02: Addiction	• Includes alcohol, soft drugs (e.g., cannabis), hard drugs (e.g., heroin, cocaine, speed, LSD), and other addictions such as gambling, sex, internet, or medication. Considering the first three categories, assessment focus is on the frequency of the infringing consumption. If the controlled use of substances is part of the treatment, then it is not seen as infringing consumption.
K03: Impulsivity	• The extent to which the patient behaved in an unpredictable and/or thoughtless way at the expense of himself or others ("first act, then think"). It can be expressed as irascibility (to have a short fuse), incontrollable immediate need-satisfaction desires (impulse-buys), or a chaotic manner of living.
K04: Antisocial behavior	• The extent to which the patient pursued his own desires and needs without either taking others' feelings and needs or the circumstances into account and the extent to which this this resulted in transgressive behavior towards others.
K05: Hostility	• The extent to which the patients attributed hostility to others, along with systems and authorities. Hostility can be manifested in the form of violent perceptions, passive aggression, cynicism, and annoyance, and sometimes in the form of severe verbal and physical aggression.
K06: Violation of terms and agreements	• The extent to which the patient adhered to his terms and agreements of his treatment. This includes the severity as well as the frequency of potential breaches of treatment agreements, the institution's rules, or furlough conditions.

K07: Influence of risky network-members	• The extent to which the patient allows himself to be influenced by others in a negative way, including fellow patients, family, friends etc. Risky networks can influence the patient to engage in risky situations or risky behavior.
Protective factors	
K08: Problem insight	• The extent to which the patient was aware of his individual risk factors and signals of risky behaviors in situations which can lead to his relapse, and the extent to which the patient behaves based on his awareness and knowledge.
K09: Social skills	• The extent to which the patient was able to maintain social contacts with his life- and work-environment in a satisfactory manner (e.g., communication skills, the proper use of manners, assertive behavior during contact with others, appropriate personal space during conversations etc.).
K10: Self-reliance	• The extent to which the patient was able to complete essential daily tasks independently, such as personal hygiene, dealing with money, patterns of diet, sleep patterns, self-presentation, care for his surroundings, and the ability to call for medial help.
K11: Cooperation with treatment	• The extent to which the patient was involved in his treatment progression. It includes patient’s cooperation, participation during treatment components, the acceptance of rules, openness for other insights and coping strategies, and the intake of prescribed medication.
K12: Crime responsibility	• The extent in which the patient accepts and takes responsibility for the offences he has committed. Some reasons for the denial of responsibility are, for instance, drug- or alcohol abuse, the fact that the offence may be committed by a group of people etc.

K13: Coping skills	• The extent to which the patient showed the correct skills to successfully resolve a confrontation with the occurrences that require adaptations (e.g., talking calmly with someone, distancing oneself from the situation, asking for help etc.).
K14: Labor skills	• The extent to which the patient was able to properly perform work or labor activities, such as work inside the clinic, a paid job, or a voluntary position with fixed tasks and hours.

Figure A1

Bootstrapped edge weights difference test at T1

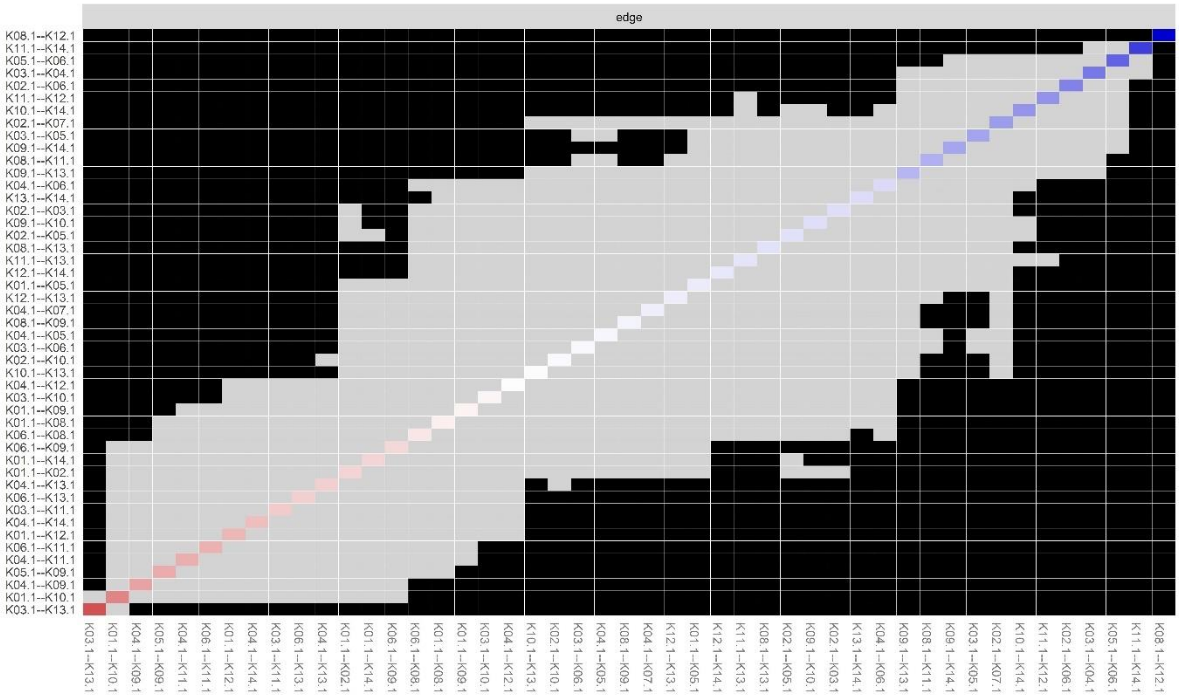

Note. The gray boxes represent edges that do not differ significantly from one-another and black boxes represent edges that do differ significantly from one-another. Colored boxes correspond to the color of the node in Figure 1.

Figure A2

Bootstrapped edge weights difference test at T2

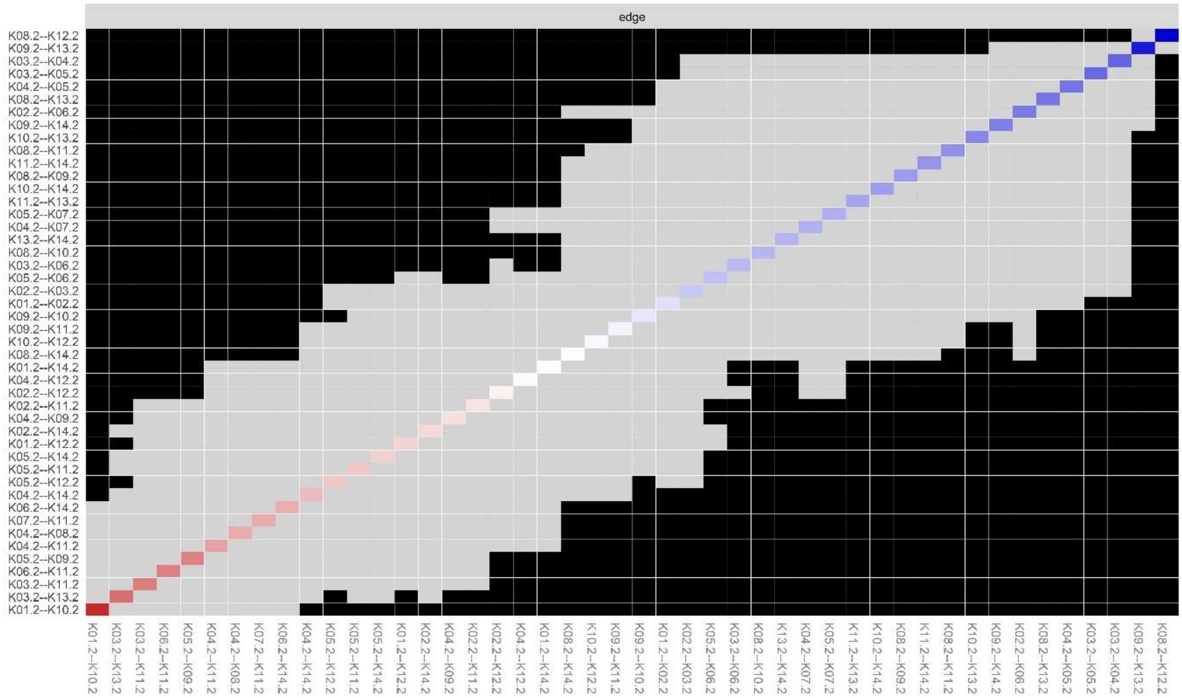

Note. The gray boxes represent edges that do not differ significantly from one-another and black boxes represent edges that do differ significantly from one-another. Colored boxes correspond to the color of the node in Figure 1.

Figure A3

Bootstrapped confidence intervals of estimated edge-weights for the estimated network at T1

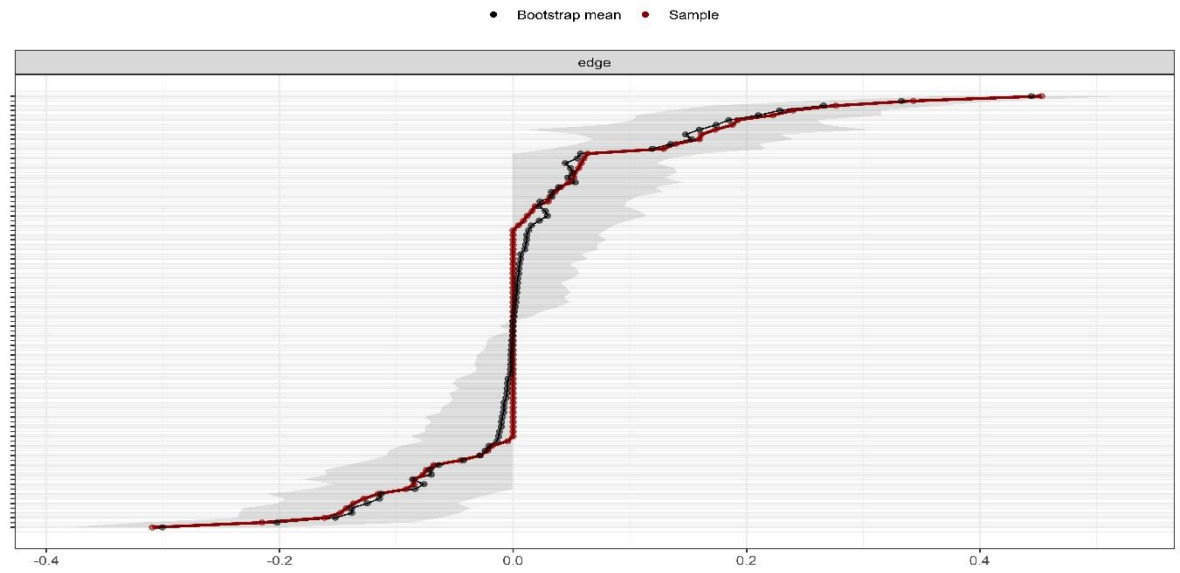

Note. Red line represents the sample values and the gray area the bootstrapped CIs. The horizontal lines indicate edges of the network, ordered from the edge with the highest edge-weight to the edge with the lowest edge-weight. The y-axis labels have been removed to avoid cluttering.

Figure A4

Bootstrapped confidence intervals of estimated edge-weights for the estimated network at T2

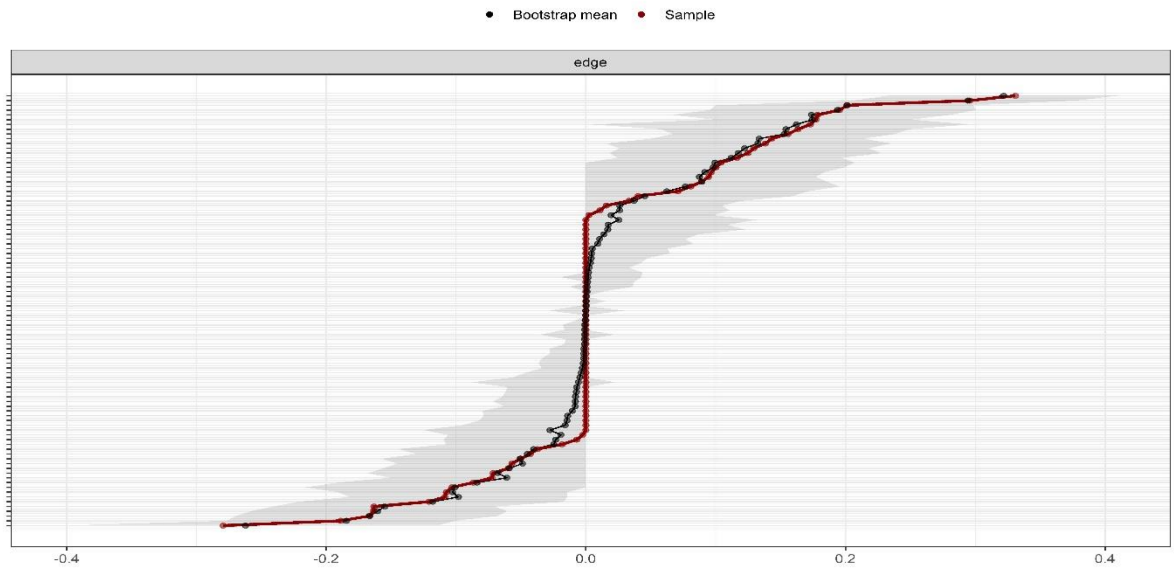

Note. Red line represents the sample values and the gray area the bootstrapped CIs. The horizontal lines indicate edges of the network, ordered from the edge with the highest edge-weight to the edge with the lowest edge-weight. The y-axis labels have been removed to avoid cluttering.

Figure A5

Average correlations between strength centrality index of networks sampled with persons dropped and the original sample at T1

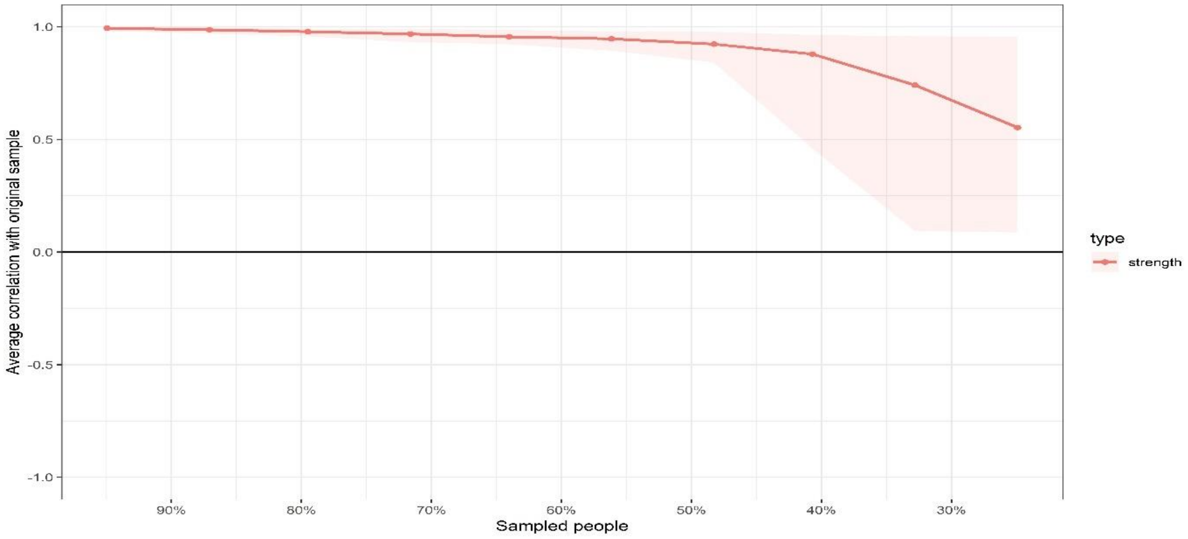

Note. Line represents the means and area indicates the range from the 2.5th quantile to the 97.5th quantile.

Figure A6

Average correlations between strength centrality index of networks sampled with persons dropped and the original sample at T2

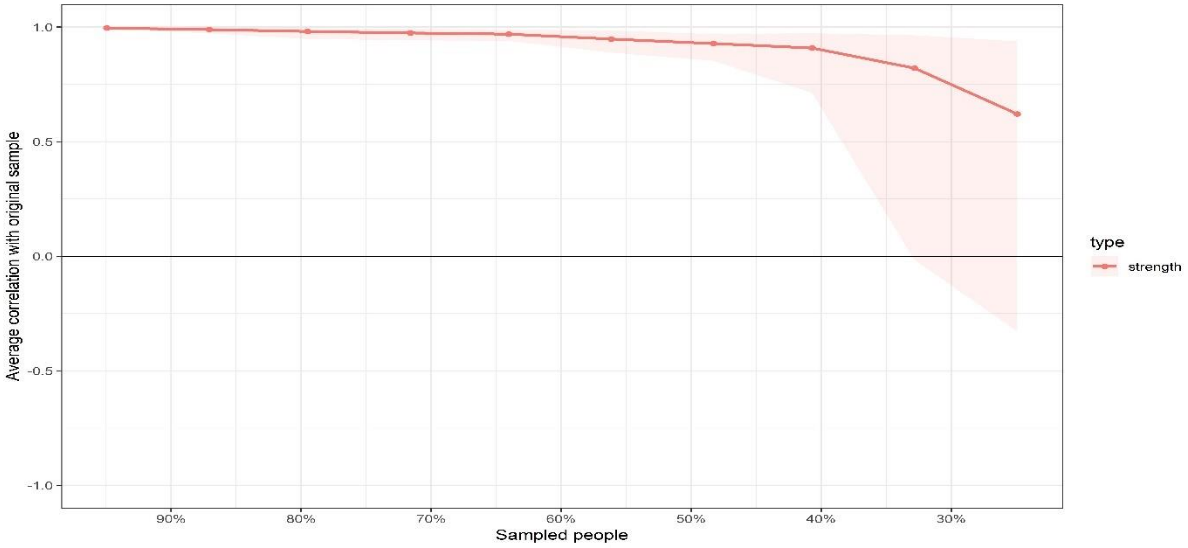

Note. Line represents the means and area indicates the range from the 2.5th quantile to the 97.5th quantile.

Figure A7

Bootstrapped strength centrality difference test at T1

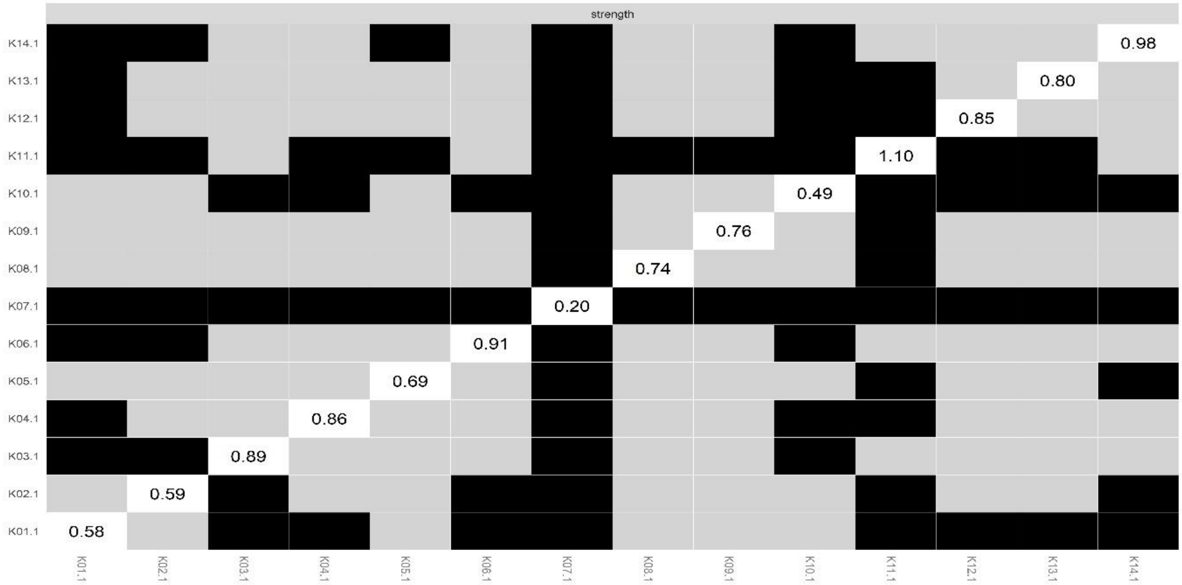

Note. The gray boxes represent nodes that do not differ significantly from one-another and black boxes represent nodes that do differ significantly from one-another. The white boxes show the value of node strength.

Figure A8

Bootstrapped strength centrality difference test at T2

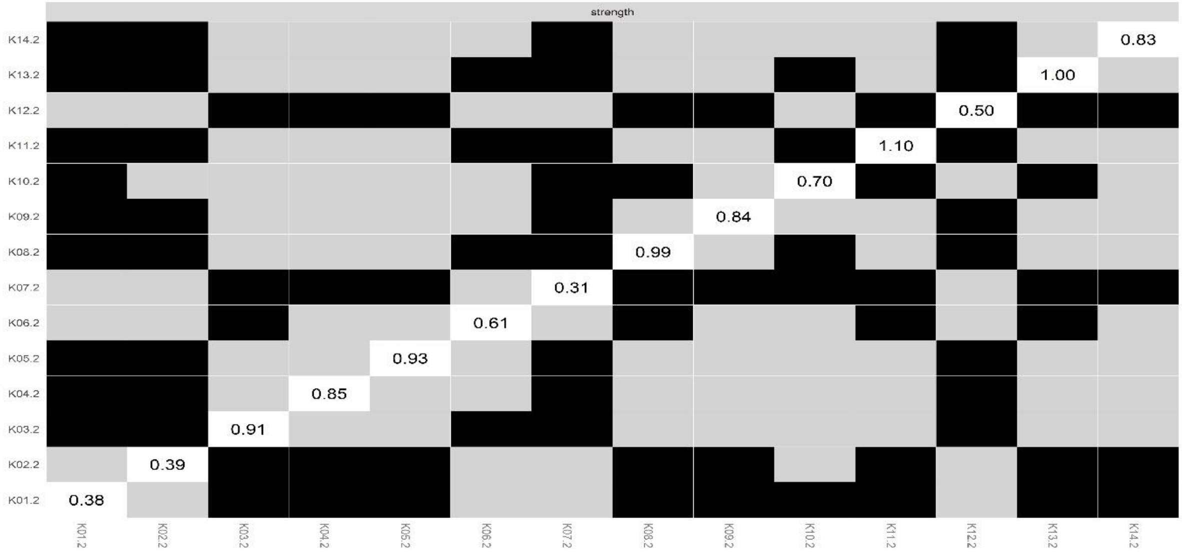

Note. The gray boxes represent nodes that do not differ significantly from one-another and black boxes represent nodes that do differ significantly from one-another. The white boxes show the value of node strength.
